# Antibacterial effect of graphene oxide (GO) nano-particles against *Pseudomonas putida* biofilm of variable age

**DOI:** 10.1007/s11356-019-05688-9

**Published:** 2019-06-27

**Authors:** Hussam Fallatah, Mohamad Elhaneid, Hanene Ali-Boucetta, Tim W Overton, Hani El Kadri, Konstantinos Gkatzionis

**Affiliations:** 1 School of Chemical Engineering, University of Birmingham, B15 2TT, Birmingham, UK; 2 School of Pharmacy, University of Birmingham, B15 2TT, Birmingham, UK; 3 Department of Food Science and Nutrition, School of the Environment, University of the Aegean, Metropolite Ioakeim 2, 81400 Myrina, Lemnos Greece

**Keywords:** Graphene oxide, *Pseudomonas putida*, Biofilm, Flow cytometry, SEM, Detached biofilm cells, Membrane damage

## Abstract

Graphene oxide (GO) has been reported to possess antibacterial activity; therefore, its accumulation in the environment could affect microbial communities such as biofilms. The susceptibility of biofilms to antimicrobials is known to depend on the stage of biofilm maturity. The aim of this study was to investigate the effect of GO nano-particles on *Pseudomonas putida* KT2440 biofilm of variable age. FT-IR, UV-vis, and Raman spectroscopy confirmed the oxidation of graphene while XPS confirmed the high purity of the synthesised GO over 6 months. Biofilms varying in maturity (24, 48, and 72 h) were formed using a CDC reactor and were treated with GO (85 μg/mL or 8.5 μg/mL). The viability of *P. putida* was monitored by culture on media and the bacterial membrane integrity was assessed using flow cytometry. *P. putida* cells were observed using confocal microscopy and SEM. The results showed that GO significantly reduced the viability of 48-h biofilm and detached biofilm cells associated with membrane damage while the viability was not affected in 24- and 72-h biofilms and detached biofilm cells. The results showed that susceptibility of *P. putida* biofilm to GO varied according to age which may be due to changes in the physiological state of cells during maturation.

**Electronic supplementary material:**

The online version of this article (10.1007/s11356-019-05688-9) contains supplementary material, which is available to authorized users.

**Figure:**
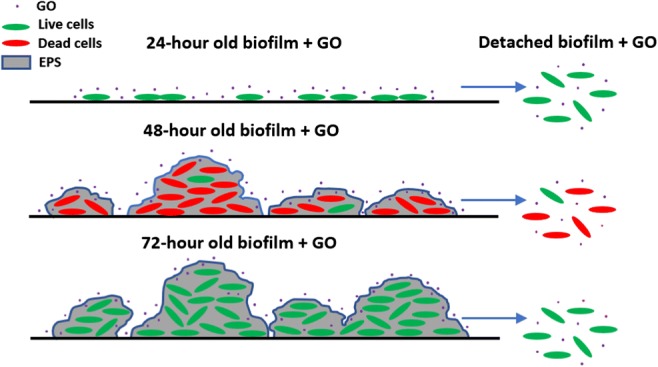
Graphical abstract

## Introduction

Graphene is a two-dimensional material and a single layer of monocrystalline graphite with sp2 hybridised carbon atoms (Jamialahmadi et al. 2018). Since its discovery in 2004, many derivatives of graphene-based nano-particles, such as graphene oxide (GO) and reduced graphene oxide (rGO) have gained interest due to their mechanical and antimicrobial properties and relatively low manufacturing cost. These have shown promising applications in various areas in industry used as metal free catalysts for degradation of organic matters in water and can be used in polymer membranes for water purification and medical cloths for wound disinfection, and also coating of silicon tubes of catheters to prevent biofilm formation (Wang et al. 2013; Zheng et al. 2018). Such applications may increase the release of non-biodegradable GO into the environment posing risks of biological toxicity (Guo and Mei 2014).

In comparison to rGO, GO contains more reactive groups that interact strongly with bacteria by covering cell surfaces, increasing its antibacterial activity (He et al. 2015a; Liu et al. 2011; Liu et al. 2012; Hui et al. 2014; Gurunathan 2015). Previous mechanisms suggested for the antibacterial activity of GO include oxidative stress (Mangadlao et al. 2015; Perreault et al. 2015), trapping of bacterial cells within GO sheets (Yadav et al. 2017), cell membrane damage by the sharpened edges of GO sheets (Gao et al. 2017), and electron transfer interaction from microbial membranes to GO (Li et al. 2014). Nevertheless, there is contradiction in the literature as some studies suggested that GO has no antibacterial activity (Bianco 2013).

In nature, bacteria attach themselves to surfaces to form communities known as biofilms by generating extracellular polymeric substance (EPS) consisting of polysaccharides, proteins, and nucleic acids. In the environment, biofilms actively participate in organic matter decomposition and biogeochemical cycling, being a key component of ecosystem functioning (Balcázar et al. 2015). Since environmental biofilms affect crucial ecosystem processes, it is important to understand the effect of GO accumulation on biofilm growth and survival. In aqueous environments, GO can be sustained as homogenous colloidal suspensions (Zhao et al. 2010). Studies have reported that GO decreases bacterial activity and viability in soil (Jamialahmadi et al. 2018; Gurunathan 2015). The presence of GO in activated sludge reduced the metabolic activity and viability of bacteria and inhibited their essential microbial functions needed in activated sludge processes such as removal of organic matter and nutrients including nitrogen and phosphorus (Ahmed and Rodrigues 2013). These studies showed that GO has a negative impact on environmental bacteria.

There are four main stages that occur dynamically during biofilm formation: planktonic cells; attachment (reversible and irreversible); maturation (micro- and macro-colonies along with development of EPS); and dispersion (Monds and O’Toole 2009). Cells in each stage are physiologically different to cells in the other stages (Bester et al. 2005). Moreover, biofilms at different stages of maturity showed different susceptibility to antibiotics (Tré-Hardy et al. 2009), sanitisers (Shen et al. 2011), and silver nano-particles (Thuptimdang et al. 2015).

The presence of EPS makes the biofilm resistant to detergents, antibiotics, and other chemicals as it protects the interior of the community (Hall-Stoodley et al. 2004). It was shown that the removal of the EPS matrix enhances the antibacterial activity of rGO against *Escherichia coli* biofilms suggesting that the presence of EPS protects the cells (Guo et al. 2017). However, there is controversy in the literature regarding the effects of GO on biofilms. For example, the growth of *E. coli* biofilms was inhibited on GO coated surfaces (Carpio et al. 2012; Yadav et al. 2017). In contrast, Guo et al. (2017) reported that the formation of *E. coli* biofilm was enhanced in the presence of GO. *E. coli* and *Bacillus subtilis* biofilm formation was encouraged at low concentrations of GO while it was inhibited at high concentrations of GO (Song et al. 2018). Furthermore, there are many factors that determine the antibacterial activity of GO including the size of GO sheets (Liu et al. 2012; Yadav et al. 2017), purity of GO dispersion (Barbolina et al. 2016), bacterial species (Gao et al. 2017; Yadav et al. 2017), and growth media (Hui et al. 2014).

Studies that investigated the impact of GO on biofilms were limited to GO-coated surfaces which do not reflect GO within environmental systems i.e. colloidal suspensions (Carpio et al. 2012; Yadav et al. 2017). Moreover, studies that assessed the development of biofilms in the presence of a GO colloidal-suspension have either reported no susceptibility or enhancement of biofilm growth (Ruiz et al. 2011; Guo et al. 2017). The aim of this study was to investigate the susceptibility of biofilm to GO at different stages of maturity, based on *P. putida* KT2440, a well-characterised bacterium and effective biofilm-producer found in soil and aquatic environments. High and low concentrations of GO were tested to reflect variations in accumulation.

## Experimental

### Materials

Graphite flakes (catalogue number: 332461), sodium nitrate (NaNO_3_) > 99%, sulphuric acid (H_2_SO_4_) 95.0–98.0%, potassium permanganate (KMnO_4_) > 99% and hydrogen peroxide solution (H_2_O_2_) 30% (*w*/*w*) were purchased from Sigma-Aldrich (UK). Phosphate buffered saline (PBS) pH 7.45, tryptic soy agar (Oxoid Ltd. CM0131), and broth (Oxoid Ltd. CM0129) were purchased from Fisher Scientific (UK). Stains SYTO 9 and propidium iodide (PI) were purchased from Thermo Fisher Scientific (UK).

### Synthesis of GO from graphite

GO was prepared by a modified Hummers’ method (Hummers and Offeman 1958) as described by Ali-Boucetta et al. (2013) with modification in incubation times. Briefly, 0.2 g of graphite flakes were mixed with 0.1 g of sodium nitrate (NaNO_3_) and 4.6 mL of sulphuric acid (H_2_SO_4_) in an ice bath (< 5 °C) and stirred for 20 min at 150 rpm. Potassium permanganate (KMnO_4_, 0.6 g) was added gradually to the mixture and the resultant reaction mixture was taken out from the ice bath and kept at room temperature while stirring for 30 min at 100 rpm until the mixture started thickening and turned into a dark green paste. A volume of 9.2 mL of de-ionised water was slowly added, and the paste was mixed. The temperature rapidly raised to 50 °C, using a hot plate to maintain temperature around 95 to 100 °C for 60 min followed by further dilution with 28 mL of warm de-ionised water. The mixture was treated with 3 mL of 30% hydrogen peroxide (H_2_O_2_) to stop the oxidation process (Huang et al. 2011) and reduce the residual permanganate, manganese dioxide, and manganese heptoxide to soluble manganese sulphate. After the dispersion was treated, it was left to settle until a yellow tint is developed.

The purification process was based on several centrifugation steps. The mixture was washed with 20 mL of warm de-ionised water and vortexed and centrifuged at 12,700×*g* for 25 min at 40 °C. After centrifugation, the pellet was separated from the supernatant by decantation and this process was carried out twice. The pH of the supernatant was checked using pH paper after each wash, as it gradually become less acidic and colourless. When the discarded supernatant reached pH 3–4, the washing step was repeated with de-ionised water, centrifuged at 12,700*×g* for 25 min at 20 °C. When the supernatant reached pH > 6, GO appears in the form of a brown/golden viscous layer on top of the oxidation by-products.

To collect the GO from the pellet, warm water was added gently, and the tube was shaken to suspend GO. This step was repeated 5 times minimum until the whole GO layer was collected. A cell strainer (100 μm) was used to remove impurities in the GO suspension. The concentration of GO in the brown/gold layer was determined by transferring 1 mL into a glass vial and drying at 40 °C for 48 h to obtain the weight of the dry product; the GO concentration in the suspension was detected. The single layer sheet of GO was achieved by sonication using a water bath sonicator (80 kHz) for either 10 or 120 min at ambient temperature.

### Transmission electron microscopy

The lateral size of GO sheets were measured using TEM. A volume of 5 μL of GO (10 μg/ml) was placed on a grid covered with support of Formvar/carbon film, air dried, and examined under a TEM (JEOL 2100EX model) to obtain higher resolution images with an operating voltage ranging from 40 to 120 keV using an LaB6 filament. The lateral sizes distributions of > 60 GO sheets were measured using the ImageJ software.

### Atomic force microscopy

Atomic force microscopy (AFM) was used for characterising the morphology and thickness of individual GO sheets. A volume of 10 μL of the sonicated GO dispersion was transferred onto a silicon surface (due to its smooth surface) and dried at 40 °C for 20 min and the surface morphology of GO was characterised using an AFM (NanoWizard II AFM (JPK, UK) in contact mode at a 512 × 512 scanning resolution by JPK Nano-wizard 2 software, with a scan area of 20 μm × 20 μm.

### UV-visible spectrophotometer

UV-visible (UV-vis) spectrophotometer was used in order to determine the absorbance and spectra of GO dispersion. GO dispersion was diluted ten times with de-ionised water before measuring the absorbance. The UV-visible absorption spectra were recorded on a Varian Cary 50 Bio spectrophotometer equipped with an integrating sphere accessory for diffuse reflectance spectra over a range of 200–900 nm by using BaSO_4_ as the reference. The collected data was then analysed using the Cary Win UV software.

### Raman spectroscopy

Raman spectroscopy was used to identify the chemical properties of GO. The Raman spectra of GO was recorded after preparing the aqueous dispersions and dried on a microscope glass by evaporating the solvent in oven. Results were recorded by × 100 objective and excitation was provided by a He-Ne 633-nm laser using Renishaw inVia Raman spectrometer with WIRE 3.1 software and an average of 3 locations were measured.

### Zeta potential

The zeta potential of the GO dispersion was measured. The GO dispersion was diluted in de-ionised water (1:10) and 1 mL of the dispersion was injected in a universal folded capillary cell (Model DTS 1070, Malvern Instruments Ltd, UK) equipped with platinum electrodes and a folded capillary, ensuring that air was removed. The electrophoretic mobility (EM) at 150 V of the suspended soil particles was then measured at 25 °C using Malvern ZetaSizer Nano ZS (Malvern Instruments Ltd, UK), which uses the scattering of incident laser light to detect the soil particles at relatively low magnification. The instrument was calibrated using the zeta potential (ζ-potential) transfer standard (DTS1235) which has a ζ-potential of − 42 mV ± 4.2 mV. The mobility of the soil particles under the applied voltage was converted to the ζ-potential using the Henry equation and reported as the average and standard deviation of measurements made on three freshly prepared samples, with each sample analysed in triplicate.

### Fourier transform infrared spectroscopy

Fourier transform infrared (FT-IR) spectroscopy was employed to determine the degree of oxidation and to confirm the presence of GO. For the transmittance readings, the GO samples were ground and mixed with KBr at a 1:9 ratio (*w*/*w*). This mixture (0.1 g) was then compressed into a thin KBr disc under a pressure of 0.4 bar for 3 min. The disc was placed 10 min in the FT-IR spectroscopy before starting the analysis to reduce the interference of water molecule from the air. FT-IR spectra were obtained on a Nicolet 6700 spectrometer and the range of the spectrum was from 4000 to 500 cm^−1^ at a resolution of 4 cm^−1^. After running the sample, data were collected by signals and each of the peaks is characteristic of different vibrational modes on molecules.

### X-ray photoelectron spectroscopy

X-ray photoelectron spectroscopy (XPS) was used to determine the elemental contents and the C/O ratio of the GO dispersion. An aliquot of 100 μL of GO dispersion was added five times throughout a day onto a mould placed in an oven at 60 °C for 3 days to create a solid disc with dimensions of approximately 7 × 7 × 3 mm. Data were collected using an Omicron Multiprobe. The measurements were conducted in the main analysis chamber using an XM1000 monochromatic Al Kα x-ray source (hv = 1486.6 eV) at room temperature. The photoelectrons were detected using a Sphera electron analyser (Omicron Nanotechnology) using a step size of 0.5 eV and pass energies at 50 eV. Selected high-resolution core-level spectra were recorded using a step size of 0.1 eV and pass energies of 10 eV (resolution approx. 0.47 eV). All binding energies were corrected with reference to the C–C/C–H bond at 284.6 eV during data analysis.

The data was analysed with CasaXPS package and Shirley background, and were fitted according to the mixed Gaussian–Lorentzian (Voigt) line-shape components and asymmetry parameters where appropriate. Calibration of the binding energies was achieved using the Fermi edge of polycrystalline Ag sample, measured immediately before starting the measurements. The transmission function of the spectrometer was calibrated using a variety of clean metal foils to ensure compositional accuracy.

### Biofilm formation using CDC reactor

The biofilms were developed using the Centers for Disease Control and Prevention (CDC) biofilm reactor which is a reliable experimental tool for growing a standard biofilm (Goeres et al. 2005). The CDC reactor consists of growing biofilms on coupons held in rods immersed in a glass vessel and operating in a batch stage (i. e. no flow) followed by a continuous flow at a constant flow rate and a shear is generated by magnetic stirring. *Pseudomonas putida* KT2440 was maintained on TSA plates at 4 °C. Cells were transferred into 100 ml of TSB, incubated at 30 °C for 24 h shaking at 150 rpm. Stationary-phase cells were harvested by centrifugation (10,000*×g*, 10 min), washed, and resuspended in PBS to inoculate the CDC reactor.

A sterile CDC reactor (model 90-2, BioSurface Technologies, Bozeman, MT) CRB vessel containing 500 mL of sterile TSB (300 mg/mL) and 24 polycarbonate coupons was inoculated with *P. putida* cells (10^8^ CFU/mL) and stirred at 125 rpm for 24 h at 25 °C providing a batch stage for cells to attach to the coupons. The temperature was maintained at 25 °C using a water bath and a silicon tube blanket. Continuous flow was operated by connecting the vessel with a 20-L TSB (100 mg/mL) and an empty carboy for feeding and waste respectively (Goeres et al. 2005). Sterile medium was pumped into the vessel with a rate of 11.67 ± 0.2 mL/min using a peristaltic pump (Watson Marlow Inc., Wilmington, MA). Eight coupons were sampled every 24 h and each coupon was washed by gentle immersing and agitating in PBS to remove unattached cells. The removed rods were replaced with blanks to maintain hydrodynamic conditions within the bioreactor.

### Viability of *P. putida* after treatment with GO

Biofilms on coupons from the CDC reactor at 24, 48, and 72 h were incubated with either 4 mL of sterile de-ionised water (control), 4 mL 85 μg/mL graphite flakes suspended in de-ionised water, or 4 mL of 85 μg/mL or 8.5 μg/mL GO at 25 °C for 24 h shaking at 80 rpm. For cell counts, the coupons were transferred to Falcon tubes containing PBS and vortexed for 30 s and then sonicated using a water bath sonicator (80 kHz) for 30 s at ambient temperature, three times. Bacterial counts were determined in PBS using the Miles & Misra technique (Miles et al. 1938). Each dilution was plated in triplicate on TSA agar and incubated at 25 °C for 48 h and colonies were counted. Each experiment was conducted in triplicate (*N* = 3).

Τhe effect of GO was studied on planktonic cells detached from biofilms and was compared with control planktonic cells. Coupons were vortexed and sonicated as described above at 24, 48, and 72 h and the detached cells were incubated with either 4 mL of sterile de-ionised water (control) or 4 mL of 85 μg/mL GO shaking at 80 rpm for 5 days at 25 °C. For control planktonic cells, an overnight culture of *P. putida* was centrifuged at 10,000*×g* for 10 min and re-suspended in sterile de-ionised water. The final concentration of *P. putida* was adjusted to 10^7^ CFU/mL by diluting with sterile de-ionised water and incubated with 85 μg/mL GO shaking at 80 rpm for 5 days at 25 °C. The bacterial counts (CFU/mL) were determined on each day as described above and each experiment was conducted in triplicate (*N* = 3).

### Scanning electron microscopy

The morphological changes to the membrane of *P. putida* cells were observed using a SEM. Cells were mounted on a stub and coated with gold-palladium alloy (100 Å and 50 Å thickness respectively) sputter module in a vacuum evaporator in argon atmosphere in order to minimise surface charges and increase resolution. The samples were observed under a Quanta 3D FEG dual-beam FIB-SEM microscope operated at 15 kV.

### Confocal microscopy analysis

Biofilms on coupons were stained by adding 10 μL of PI (100 μM/L) and SYTO 9 (100 μM/mL) on their surface and incubated in the dark for 5 min. The samples were gently covered with a coverslip and were observed at room temperature using a Leica SPE-II confocal microscope (Leica) equipped with solid-state lasers for excitation. Images were acquired under × 60 magnification oil immersion objective lens with a Leica DFC500 camera and Leica LAS AF software at a 1-μm interval through the biofilms and five image stacks, each representing a different field of view on the coupon. Samples were excited using a 488 nm laser and fluorescence was detected at 617 nm (PI) and 503 nm (SYTO 9). The thickness (μm) of biofilms was measured using the Leica LAS AF software. The confocal images were Z-stacks of optical sections using 512 × 512-pixel resolution tagged image file format. From the Z-stacks of the 3D biofilm structure the biomass volume (μm^3^ / μm^2^) was calculated using COMSTAT software (Heydorn et al. 2000; Vorregaard 2008) and the percentage of live/dead cells was determined.

### Assessment of *P. putida* membrane integrity with flow cytometry analysis

Biofilms on coupons from the CDC reactor at 24, 48, and 72 h were incubated with either 4 mL of sterile de-ionised water (control) or 4 mL of 85 μg/mL GO at 25 °C for 24 h shaking at 80 rpm. To obtain a dispersed cell suspension from biofilms, the coupons were transferred to Falcon tubes containing PBS and vortexed for 30 s and then sonicated using a water bath sonicator (80 kHz) for 30 s at ambient temperature, three times. Dispersed *P. putida* cells (1 mL) were stained with PI to a final concentration of 4 μL/mL and incubated in the dark for 5 min. Flow cytometric analysis was conducted using the BD Accuri C6 flow cytometer (BD Biosciences, UK) and samples were excited using a 488-nm solid-state laser and particulate noise was eliminated using a Forward scatter height (FSC-H) threshold while 20,000 data points were collected at a maximum rate of 2500 events/s. PI fluorescence was detected using 670 LP filters and data was analysed using the CFlow software. Each experiment was conducted in triplicate (*N* = 3).

### Statistical analysis

The generated results were collected in Excel (Microsoft Corp.) for calculating means, standard deviations, and error bars. Student’s *t* test to compare two means or one-way analysis of variance (ANOVA) and Tukey’s HSD post hoc test to compare several means were used for checking whether there is a significant difference among samples using the IBM SPSS Statistics software version 23. Differences were considered significant at *P* < 0.05.

## Results and discussion

### GO characterisation

The GO dispersion had a pH ~ 6.5 and the achieved concentration 117 ± 10 mg/mL was higher than the yield achieved by Jasim et al. (2016) and Ali-Boucetta et al. (2013) although lower than Frankberg et al. (2015). This variation depends on the source of graphite used to synthesise GO (Jasim et al. 2016) and oxidation time (Huang et al. 2011). The GO sheets were stable and maintained solubility in water (Fig. S1) and this is mainly due to their numerous oxygen-containing functional groups. Raman spectroscopy was employed as a non-destructive technique to study the bonding nature of GO and graphite (Fig. S2). The main features in Raman spectra for carbon materials are the D- and G-band and according to literature are around the wavenumber of 1360 and 1560 cm^−1^, respectively (Sur et al. 2016; Khan et al. 2017). The occurrence of the G-band is due to the first order scattering of the E_2g_ mode and the “D-band” associates with the defects in the graphite lattice. Results showed a symmetry structure for GO and graphite. The Raman spectrum of GO showed the presence of a G-band at 1583.12 cm^−1^ and a D-band at 1328.63 cm^−1^. The G-band in GO was shifted toward a higher wavenumber; this is due to the oxygenation of graphite which results in the formation of sp3 carbon atoms. The D band in the GO was broadened due to the reduction in size of the sp2 domains during oxidation. Also, the D-band increases in intensity in GO, indicating the successful oxidation of the graphite structure (Perreault et al. 2015). Graphite exhibits a 2D-band near 2681 cm^−1^ which disappeared in GO. It has been reported that the 2D-band correspond to the number of layers of graphene sheets and their relative orientation (López-Díaz et al. 2017). The results of Raman spectrum analysis of graphene show significant changes compared with the spectrum of GO and are similar to those reported by other studies (Ali-Boucetta et al. 2013; Wei et al. 2017; Gao et al. 2017) which indicates that graphite has been oxidised.

**Fig. 1:**
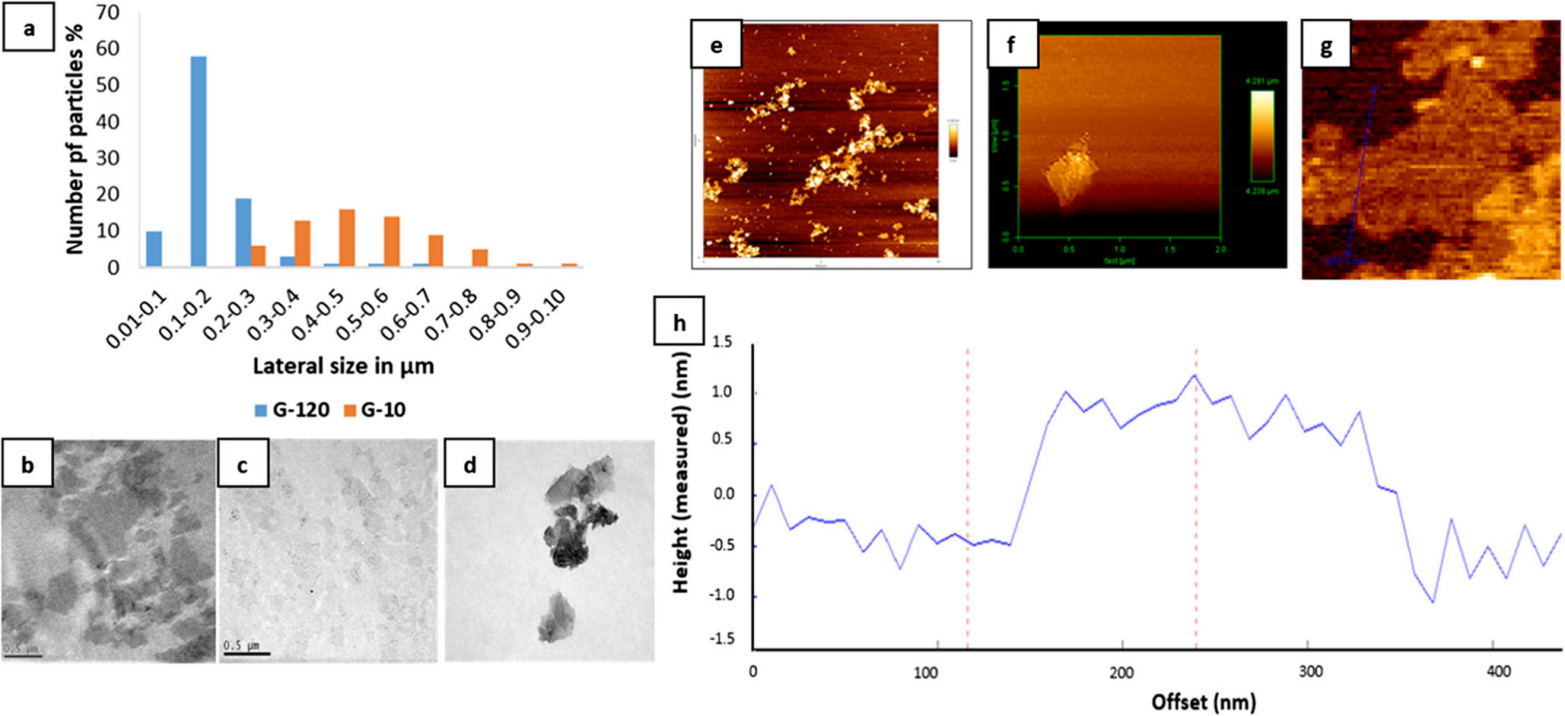
Lateral size in micrometres and height (thickness) in nanometres of GO sheets using TEM and AFM, respectively. Size distribution of GO sheets sonicated for 10 min (G-10) and for 120 min (GO-120) using TEM (**a**). TEM images of GO-10 (**b**), GO-120 (**c**), and wrinkled GO (**d**). Topographical images of GO-120 sheets acquired using AFM (**e**–**g**). Top view of GO sheets on silicon wafer (**e**), sharp edges of single GO sheet (**f**) and a cross-section analysis along the cross line of GO (**g**) to measure the height of GO from the silicon wafer surface. The corresponding height (in nm) of GO-120 sheets at the bottom (**h**)

In this study, two different GO dispersions were formed by sonication for 10 min (GO-10) or 120 min (GO-120). The morphology of GO conjugates was characterised using TEM and AFM. As expected, the GO sheets were smaller in GO-120, and 63% had a lateral size of < 200 nm (Fig. 1a–c). GO-120 showed flake-like structures due to the irregular boundaries of the GO sheets. Furthermore, these structures exhibited few-layered 2D configuration with typical wrinkled GO sheets (Fig. 1d). Based on these results GO-120 was selected for the antibacterial studies and therefore further characterised. Under AFM, GO-120 also showed a sheet-like morphology with sharp edges (Fig. 1e–g) and the thickness of the GO sheets range from 1.6–2 nm suggesting the formation of a single-layer 2D GO nanosheet (Fig. 1 h). Studies have reported thinner GO sheets (Huang et al. 2011; He et al. 2015a; Mukherjee et al. 2016; Rathnayake et al. 2017; Chu et al. 2018); however, this is possibly due to prolonged graphite exfoliation which reduces the thickness of GO sheets.

ζ-potential was employed to characterise the stability of colloidal dispersions and the magnitude and sign of the effective surface charge connected with layer around the colloid particle. The ζ-potential of GO-120 was − 36.4 mV (Fig. S3a) which indicates good stability of the aqueous dispersion of GO sheets. In colloidal dispersions, particles with less than − 30 mV or more than + 30 mV are considered as stable dispersions due to interparticle electrostatic repulsion (Konkena and Vasudevan 2012). The GO dispersions had a highly negative charge because of the oxygen group added on the surface of GO sheets. The ζ-potential was calculated using the Henry equation with the electrophoretic mobility (Sur et al. 2016) that assumes spherical shape objects; therefore, caution is advised in use of the exact ζ-potential values (Jasim 2016). Also, no signs of agglomeration or coagulation in water were observed within 6 months of storage at ambient temperature (Fig. S3b). The high solubility of the GO sheets in water could be due to their numerous oxygen-containing functional groups (Liu et al. 2011). The presence of oxygen-containing functional groups on the sheet basal plane and sheet edge of GO makes it highly hydrophilic (Kashyap et al. 2014). This suggests that the acid treatment places oxygen-containing functional groups on the graphene surface (Manafi et al. 2017).

The UV-vis spectra of the GO showed a strong absorption peak at 235 nm and a weak peak around 300 nm (Fig. S4). The sharp peak at 235 nm is due to the 휋-휋* transition of aromatic C=C bonds and the weak broad absorption band (shoulder peak) around 300 nm shows the 휋-휋* transitions of C=O bonds. FT-IR was used to measure the adsorptions of GO-120 dispersion and determined different types of oxygen functionalities present (Fig. S5). The FT-IR graph of GO-120 shows the stretching of hydroxyl group at 3405 cm^−1^ i.e., the O–H stretching vibrations of the C–OH groups. The peak at 1729 cm^−1^ refers to a carbonyl group (C=O) and the peak at 1383 cm^−1^ represents C–OH stretching groups. In the aromatic region around 1500–1650 cm^−1^, there were two peaks at 1590 and 1640 cm^−1^ that are related to aromatic rings with no substitution (Jasim et al. 2016).

XPS surface analysis was used to determine the chemical contamination, the ratio of carbon and oxygen C:O, and the quantification groups in GO sample (Fig. S6). The GO-120 scan shows two main elements: carbon C1s around 284.0 eV and oxygen O1s around 533 eV (Fig. S6a). The scan also shows the atomic compositions and the concentrations of different elements. Carbon and oxygen were the major elements at 68.85 and 30.54%, respectively. Therefore, the ratio of carbon to oxygen was 2:1. Sulphur (sp2) and nitrogen (N1s) were also detected on GO-120 surfaces at 0.48 and 0.13%, respectively. The presence of sulphur and nitrogen in very low concentrations was due to the purification and centrifugation steps that remove impurities and increase the purity of the GO dispersion (Barbolina et al. 2016). The carbon chemical states show information about the surface chemical composition of GO. The lower binding energy at 283.5 eV represents the sp2 carbon bond C–C in aromatic rings (Cheng et al. 2013). The C1s peak resolved into four peaks (or chemical states) including hydrocarbon groups C–C/C–H, C=C, carbonyl groups C=O and O–C=O as shown in Fig. S6b. The curve-fitting process was achieved by assigning the peaks to their corresponding binding energies of their suspected chemical states reported in the literature (Beamson and Briggs 1992). The double bonds between carbons C=C are shown at 284.6 eV while the carbon peaks at 286.7 and 288.3 eV represent C=O and O–C=O, respectively. These assignments agree with reference (Cheng et al. 2013; Sarawutanukul et al. 2018). The C=O groups mainly arise from ketones which are present on edges of GO sheets but may also be bound to the sheets as carbonyl groups (Song et al. 2014) were the highest on the GO surfaces followed by C=C. The C=O group derived from the epoxy (–O–) group on the surface due to the attachment of oxygen from water molecules (Sarawutanukul et al. 2018). Results show that GO samples have organic compounds with multiple functional groups. The O1s spectra in Fig. S6c shows information about the oxygen states on the surface of GO. Three components were found at 532.3, 531.2, and 533.3 eV that correspond to COO (531.2 eV), C=O (532.2 eV), and OH (533.3 eV). These assignments agree with those previously reported (Geng et al. 2013). The OH could be due to water vapour that covers the sample surface from the environment or may be attached to GO-120. These results show that oxygen is effectively interacted with graphene and suggest that the GO-120 sheet contains large numbers of functional groups on the surface such as COO and C=O, which confirms the FT-IR data. Finally, XPS showed that the oxidation led to an increase of oxygen functionalities in the form of carboxylic groups and ketones.

### Effect of GO on *P. putida* biofilm

**Fig. 2:**
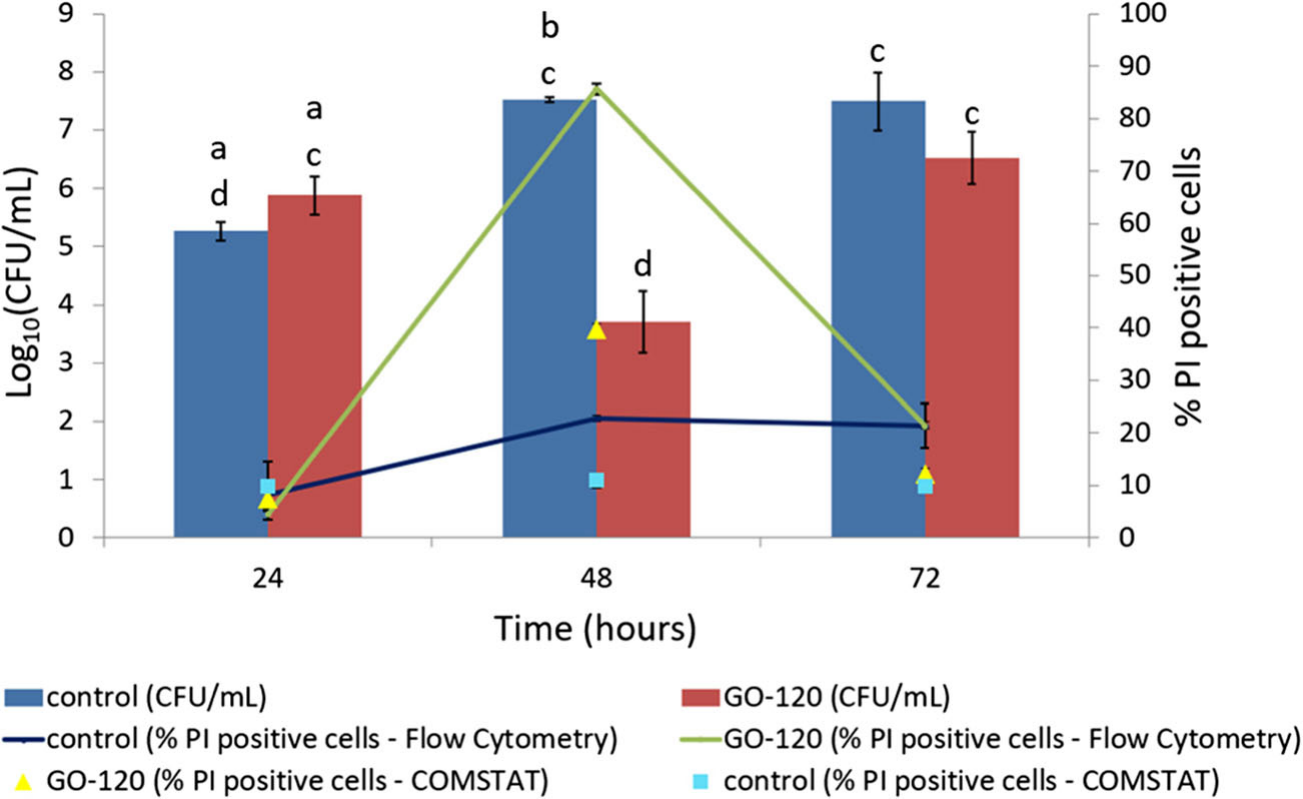
*P. putida* growth in CFU/mL (bars) and percentage of PI positive cells in biofilms incubated with GO-120 (85 μg/mL) for 24, 48, and 72 h measured using flow cytometry and confocal microscopy with COMSTAT analysis. Bars represent mean ± SEM taken from a minimum of 3 independent experiments. Mean values with different letters are significantly different (*P* < 0.05). The data was analysed with one-way ANOVA

The activity of GO against 24-, 48-, and 72-h mature *P. putida* biofilms was monitored by plate counting (CFU/mL) (Fig. 2). Bulk graphite showed no effect on viability of *P. putida* biofilms (Fig. S7). These results correlate with a study by Liu et al. 2011 reporting that bulk graphite (400 μg/mL) has no antibacterial effects against planktonic *E. coli*. There was no significant difference in viability of *P. putida* biofilm treated with GO after 24- and 72-h development compared with controls; however, the viability at 48 h was significantly (*P* < 0.05) lower indicating an age dependency of the biofilm susceptibility to GO.

Flow cytometry and confocal microscopy were employed to assess the membrane integrity of *P. putida* cells as an indication of cell damage or death after treatment with GO. PI is a membrane-impermeable dye and is commonly used to stain cells with damaged or compromised membranes, whereby it binds to DNA and emits red fluorescence, indicative of dead cells. To establish if GO sheets would interfere with flow cytometric analysis, a solution of GO was stained with PI and analysed by flow cytometry to measure the background noise. The percentage of GO sheets detected at a concentration of 85 μg/mL and 8.5 μg/mL were 3.1–1.3% and 0.4–0.06%, respectively (showing that background noise was too low and should not interfere with the analysis of cells. In controls, the percentage of PI-positive cells was 2.8-fold higher in 48- and 72-h biofilms compared with 24-h biofilm (Fig. 2). Flow cytometry data revealed that after treatment with GO, the percentage of PI-positive cells was 18.8-fold higher in 48-h *P. putida* biofilm compared with 24-h *P. putida* biofilm and 4.4-fold higher compared with 72-h *P. putida* biofilm. Furthermore, in 24- and 72-h biofilms, the percentage of PI-positive cells was similar in the control and after GO treatment, but in the 48-h biofilm, the percentage of PI-positive cells was 4-fold higher after GO treatment compared with the control. These results indicate that the antibacterial activity of GO on *P. putida* depends on the maturity stage of the biofilm.

Confocal microscopy showed an increase in PI intensity after GO treatment for biofilms at 48 h compared with 24 and 72 h (Figure 7A and S7A). The image stacks of the 3D biofilm structure were analysed using the COMSTAT software to obtain the percentage of live/dead cells (Fig. 6 and Table S1). In the control, the percentage of PI-positive cells was similar in all samples. After GO treatment, the percentage of PI-positive cells in 48-h *P. putida* biofilm was 5.2- and 3.3-fold higher than that in 24- and 72-h *P. putida* biofilms, respectively. Furthermore, in 24- and 72-h biofilms, the percentage of PI-positive cells was similar in the control and after GO treatment, but in 48-h biofilms, the percentage of PI-positive cells was 3.6-fold higher after GO treatment compared with the control. These results further confirm flow cytometry and CFU data showing an increase in numbers of dead cells that only occur in 48-h mature *P. putida* biofilm after GO treatment. Although there is a correlation between flow cytometry and COMSTAT data, the percentage of PI-positive cells for GO-treated and control biofilms obtained using flow cytometry were nearly double the percentages obtained by COMSTAT. This discrepancy can be attributed to the high amounts of SYTO 9 used for confocal imaging to reduce its replacement by PI at nucleic binding sites which falsely increases the green fluorescence/red fluorescence ratio used to determine viability (Lehtinen et al. 2004). PI competes with SYTO9 for nucleic binding sites, which may lead to the release of bound SYTO9 from DNA. PI competes with SYTO9 for nucleic binding sites, which may lead to the release of bound SYTO9 from DNA.

**Fig. 3:**
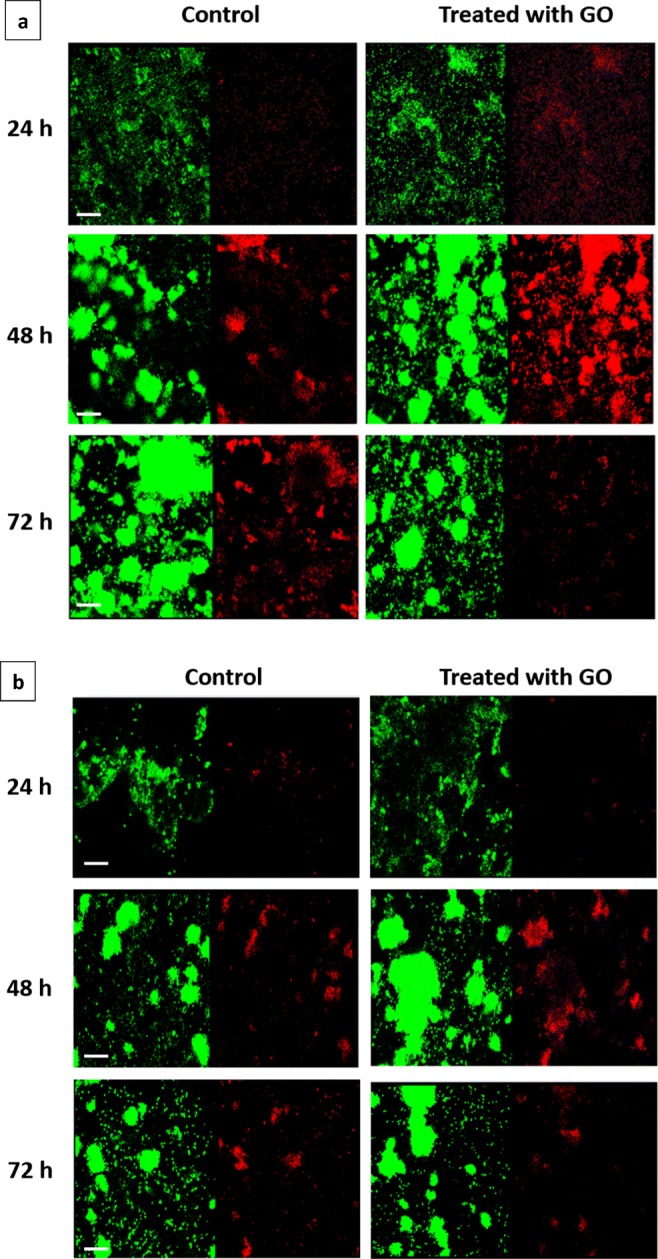
Confocal images (top view) of *P. putida* biofilm with or without GO-120. 85 μg/mL (**a**) and 8.5 μg/mL (**b**) at 24, 48, and 72 h. The biofilms were stained with SYTO 9 (left) and PI (right). Scale bar: 20 μm

Confocal microscopy showed an increase in PI intensity after GO treatment for biofilms at 48 h compared with 24 and 72 h (Fig. 3a). The image stacks of the 3D biofilm structure were analysed using the COMSTAT software to obtain the percentage of live/dead cells shown in Fig. 2. In control, the percentage of PI-positive cells was similar in all samples. After GO treatment, the percentage of PI-positive cells in 48-h *P. putida* biofilm was 5.2- and 3.3-fold higher than that in 24- and 72-h *P. putida* biofilms, respectively. Furthermore, in 24- and 72-h biofilms, the percentage of PI-positive cells was similar in control and after GO treatment, but in 48-h biofilms, the percentage of PI-positive cells was 3.6-fold higher after GO treatment compared with the control. These results further confirm flow cytometry and CFU data showing an increase in numbers of dead cells that only occur in 48-h mature *P. putida* biofilm after GO treatment. Although there is a correlation between flow cytometry and COMSTAT data, the percentage of PI-positive cells for GO-treated and control biofilms obtained using flow cytometry were nearly double the percentages obtained by COMSTAT. This discrepancy can be attributed to the high amounts of SYTO 9 used for confocal imaging to reduce its replacement by PI at nucleic binding sites which falsely increases the green fluorescence/red fluorescence ratio used to determine viability (Lehtinen et al. 2004).

**Fig. 4:**
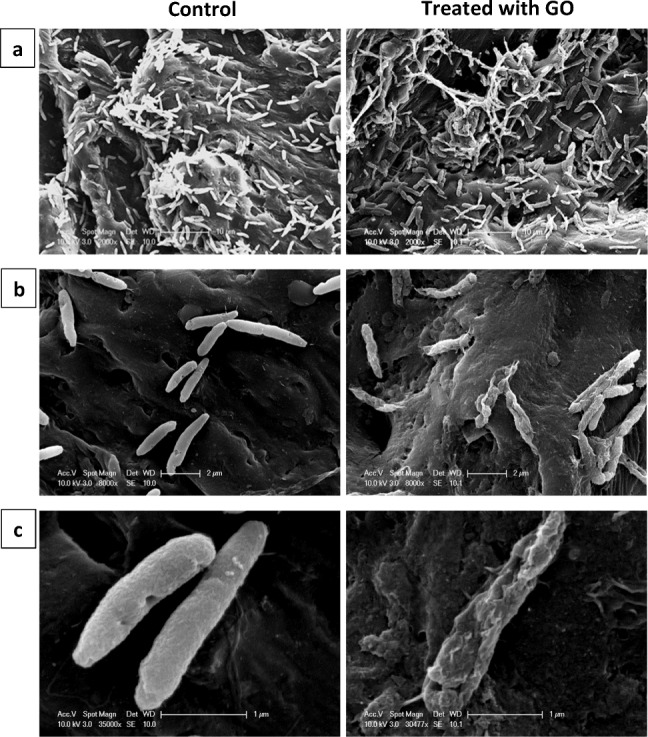
Scanning electron microscopy (SEM) images of 48-h mature biofilm of *P. putida* without (left) and with GO-120 (85 μg/mL) (right) treatment. Scale bar: **a** 10 μm, **b** 2 μm, and **c** 1 μm

To further investigate the mechanism of GO affecting the cellular membrane of *P. putida*, SEM analysis was carried out on 48-h biofilm after GO treatment (Fig. 4). Most GO-treated *P. putida* cells showed cell membrane damage while the control showed an intact cell membrane. These results suggest that the loss of cell viability was associated with membrane damage. Very few studies investigated the antibacterial activity of GO against bacterial biofilms, and contrary to our results, they suggested that GO has no effect against biofilms. In a study by Ruiz et al. (2011) GO at a concentration of 25 μg/mL was shown to promote faster growth of *E. coli* in rich LB media resulting in dense biofilm formation. They observed GO precipitation and aggregation in LB media which suggested that may be acting as a scaffold for bacterial attachment, proliferation, and biofilm formation. In a recent study, Guo et al. (2017) investigated the impact of GO against *E. coli* and *Staphylococcus aureus* biofilms in LB media. They found that GO promotes biofilm formation and development and possesses no antibacterial activity on mature 48-h biofilms with concentrations ranging from 0.5 to 500 mg/mL. However, all these studies investigated the antibacterial effects of GO in LB media. According to Hui et al. (2014), the antibacterial activity of GO (200 μg/mL) against *E. coli* was completely inhibited in LB-supplemented saline compared with saline solution due to components in LB adsorbing onto GO basal planes deactivating its antibacterial activity. In the present study, the experiments were done in de-ionised water as opposed to LB media, and therefore, it is not possible to compare the results. Also, the antibacterial effects of GO may vary against different bacterial species. Gao et al. (2017) found that the membrane integrity of *E. coli* and *S. aureus* was compromised after exposure to GO. However, the amount of RNA released from *S. aureus* was higher than that from *E. coli* which was attributed to the smaller size of the *S. aureus* which makes it more susceptible to the “cutting” and “wrapping” mechanisms of GO sheets. Yadav et al. (2017) found selectivity in antimicrobial activity of GO due to size differences; smaller GO sheets showed higher antibacterial activity against *E. coli* compared with *S. aureus* due to having a thinner cell wall which could be easily pierced by the GO sheets.

The pH of GO depends on its purity and this may affect its antibacterial activity. In a recent study, Barbolina et al. (2016) showed that adding several washing steps can change the pH of GO solution from acidic to neutral. Exposure to un-washed GO reduced the survival of *E. coli* and *S. aureus* in a concentration-dependent manner (10-250 μg/mL) while the washing steps during the purification process diminished the antibacterial effects of GO. The authors suggest that the acidic pH was responsible for the antibacterial activity of un-washed GO. However, in this study, the highly purified GO was obtained using a modified Hummers’ method in accordance to Ali-Boucetta et al. (2013) and the pH of treated samples after adding GO was ~ 6.5 which do not impact bacteria.

There is inconsistency in the literature regarding the effects of antibacterial agents against biofilms of variable age. Several studies reported that mature biofilms are more susceptible to antimicrobial agents compared with young biofilms (Anwar et al. 1989; Frank and Koffi 1990; Leriche and Carpentier 1995; Hoiby et al. 2001; Tré-Hardy et al. 2009; Shen et al. 2011; Pandit et al. 2015). Thuptimdang et al. (2015) found that 48-h *P. putida* biofilms showed higher resistance to silver nano-particles compared with 6-, 12-, and 30-h *P. putida* biofilms. In contrast, Chumkhunthod et al. (1998) found that there was no significant difference in susceptibility between 1, 2, and 3-day *P. putida* biofilm to non-foaming acidic and liquid hypochlorite sanitizers. However, in this study, it was shown that antibacterial activity of GO depended on the age of the biofilm and only occurred at a specific time during its developmental stage. Furthermore, the growth of cells and formation of EPS is also dependent on the type of media, pH, temperature, and strain of bacteria (Combrouse et al. 2013) which may explain the variability observed between the different studies.

### Effect of GO concentration, exposure time, and lateral size on *P. putida* biofilm

**Fig. 5:**
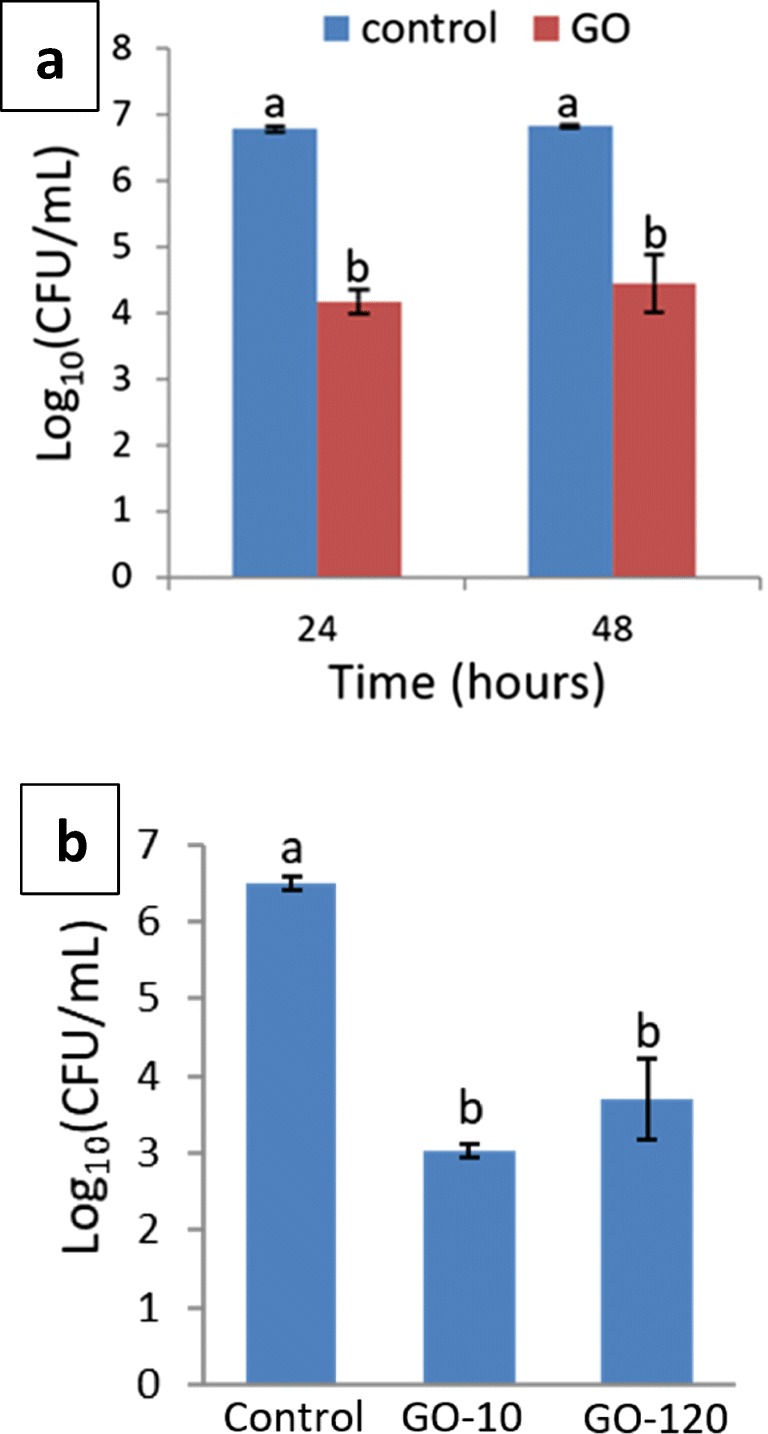
Viability of 48-h biofilm *P. putida* treated with GO (85 μg/mL) for different incubation time of 24 or 48 h (**a**) and with different sizes (GO-10 vs GO-120) for 24 h (**b**). Bars represent mean ± SEM taken from a minimum of 3 independent experiments. Mean values with different letters are significantly different (*P* < 0.05). The data was analysed with one-way ANOVA

A lower concentration of GO (8.5 μg/mL) had no antibacterial activity against 48-h biofilm (Fig. S8) and the *P. putida* membrane integrity was not compromised (Fig. 3b). These results are in agreement with studies that show a dose-dependent antibacterial activity of GO against planktonic bacteria (Liu et al. 2011; Ahmed and Rodrigues 2013; Chen et al. 2014; He et al. 2015a; Combarros et al. 2016; Gao et al. 2017). To test the effect of prolonging the time of GO exposure, the viability of 48-h *P. putida* biofilm was assessed after GO-treatment for 24 and 48 h. The viability of 48-h mature *P. putida* biofilm with GO was significantly (*P* < 0.05) lower compared with control after 24 and 48 h (Fig. 5a). However, there was no significant difference in viability of 48-h mature *P. putida* biofilm with GO at 24 h compared with 48 h; therefore, prolonging exposure to GO did not result in further reduction in viability.

In this study, the effect of varying the lateral size of GO sheets (G-10 vs G-120) had no effect on the viability of 48-h *P. putida* biofilm (Fig. 5b). These results are in contradiction to findings by Liu et al. (2012) that antibacterial activity of GO sheets against *E. coli* increased in a size-dependent manner ranging from 0.753 to 0.01 μm^2^. Similarly, Campos-Delgado et al. (2016) reported that GO sheets with larger lateral size (> 2 μm) showed higher antibacterial activity against *E. coli* compared with GO sheets with smaller lateral size (100 nm). The authors suggested that GO wraps the bacterial cells and prevents them from being able to uptake nutrients and proliferate on agar plate leading to reduced counts. Yadav et al. (2017) found a selectivity in antibacterial activity of two types of GO sheets (GO_I_ and GO_H_) prepared using different methods against *E. coli* and *S. aureus*. Since oxidative stress was found to be similar in both, it was suggested that the selectivity in antimicrobial activity of each GO was due to size differences of GO_I_ and GO_H_ (200 and 1200 nm, respectively). The authors suggested that smaller GO_I_ sheets pierce the thin cell wall in *E. coli* cells while cell wall in *S. aureus* is difficult to pierce by GO_I_, and therefore, wrapping of the bacterial cells by larger sheets of GO_H_ is the predominant mechanism of killing. However, these studies investigated the effect of GO sheets against planktonic bacteria and not biofilm. Also, the size difference between the large and small GO sheets was greater in those studies compared with this study.

It has been observed that nano-particles can penetrate and deposit in biofilms (Miller et al. 2013). Biofilm and specifically EPS enhance the retention of GO nano-particles due to the surface roughness and physical straining (He et al. 2015b). Once inside the biofilm, GO may come in contact with bacteria and induce their antibacterial activity. Several mechanisms have been suggested in regard to the antibacterial actions of GO against planktonic bacteria including “sharp” edges of the GO nanosheets cutting through the cell membrane leading to leakage and death (Chen et al. 2014; Gurunathan 2015; Nanda et al. 2016), wrapping of large GO sheets around the cells to block interactions isolating them from the environment (Carpio et al. 2012; Perreault et al. 2015) and ROS-dependent and ROS-independent oxidative stress (Ahmed and Rodrigues 2013; Li et al. 2014). In addition to the loss in cell viability, many studies reported flattening and loss of cellular structure in bacteria treated with GO (Liu et al. 2011; Chen et al. 2014; Perreault et al. 2015; Nanda et al. 2016; Combarros et al. 2016; Farid et al. 2018) which corroborate with our findings. Tu et al. (2013) showed that GO can induce the degradation of the inner and outer cell membranes of *E. coli* and reduce their viability. Using molecular dynamics simulations, the authors suggest that two types of molecular mechanisms drive the degradation of *E. coli* cell membranes: one by severe insertion and cutting and the other by destructive extraction of lipid molecules. Since in this study there were no differences in the antibacterial activity between different sizes of GO sheets, the mechanism of membrane cutting due to sharp edges is unlikely. Also, SEM images do not show GO sheets being inserted within *P. putida* membranes. Moreover, the size of the GO sheets is not large enough to wrap the cells. Therefore, the most probable mechanism would be oxidative stress, however, further investigation is required to understand which mechanism is responsible for the antibacterial activity of GO.

### Effect of GO on detached biofilm and planktonic *P. putida*

The exposure to GO-120 did not affect the thickness of biofilms measured using confocal microscopy images (Fig. S9). In a recent study by Guo et al. (2017) the removal of EPS in biofilm enhanced the antibacterial activity of rGO suggesting that EPS acts “as a barrier” to protect the cells from physical damage and “act as a sink” of ROS by limiting their damaging activity on the cells. GO may also adsorb the components of EPS and aggregate preventing it from coming in contact with bacterial cells.

**Fig. 6:**
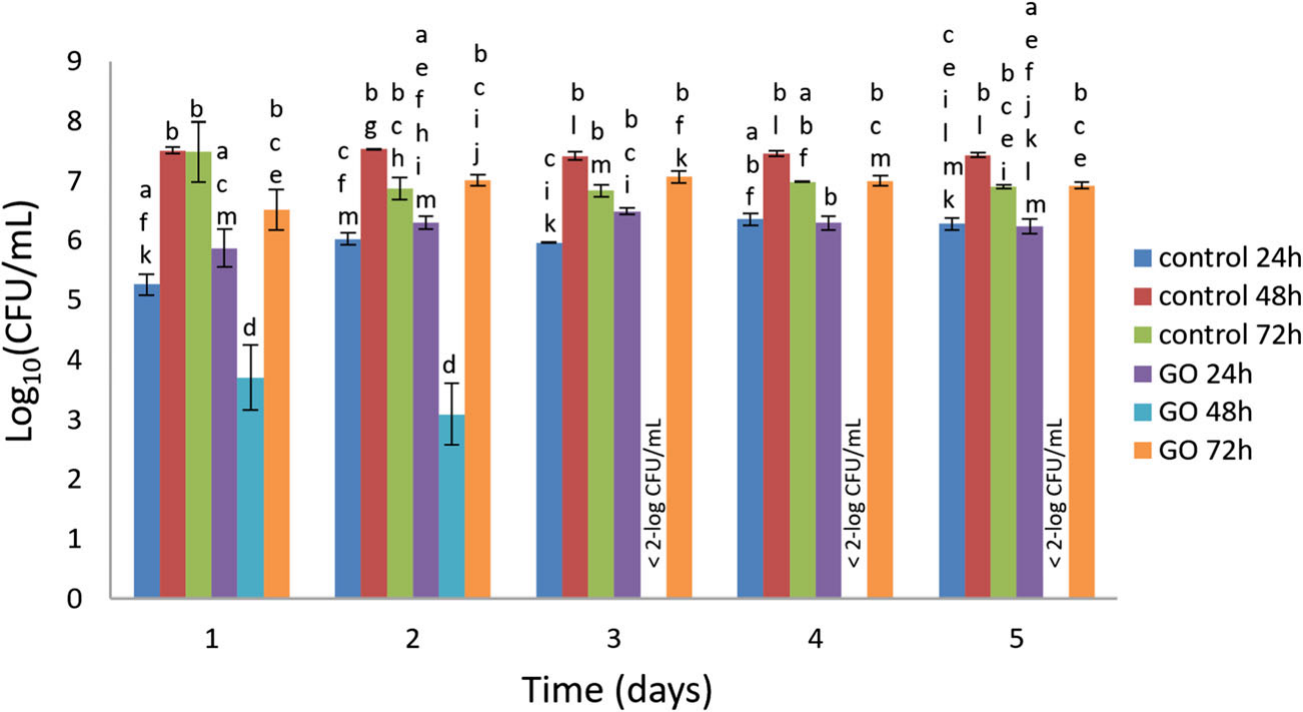
Viability of planktonic cells of *P. putida* detached from 24-, 48-, and 72-h mature biofilms and incubated with GO-120 (85 μg/mL) for 1, 2, 3, 4, and 5 days. Bars represent mean ± SEM taken from a minimum of 3 independent experiments. Mean values with different letters are significantly different (*P* < 0.05). The data was analysed with one-way ANOVA

**Fig. 7:**
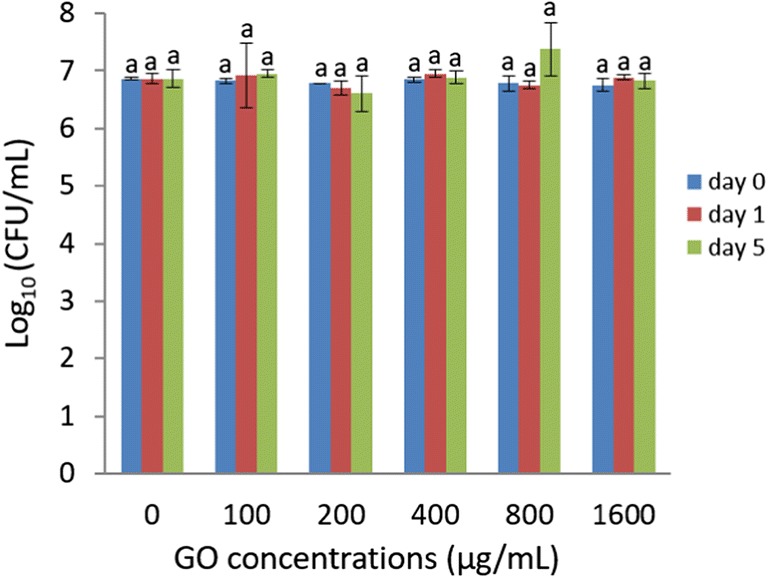
Viability of planktonic cells of *P. putida* incubated with varying concentrations of GO-120 at days 0, 1, and 5. Bars represent mean ± SEM taken from a minimum of 2 independent experiments. Mean values with different letters are significantly different (*P* < 0.05). The data was analysed with one-way ANOVA

To investigate the role of EPS in biofilm maturity-related susceptibility of *P. putida*, 24-, 48-, and 72-h detached biofilm cells were treated with GO and viability was assessed over time (Fig. 6). There was no significant difference in viability between 24- and 72-h detached *P. putida* treated with GO compared with control after 1, 2, 3, 4, and 5 days. Surprisingly, the viability of 48-h detached biofilm cells treated with GO was significantly (*P* < 0.05) lower compared with all the other samples after 1 and 2 days and was completely inactivated from day 3 onwards. Therefore, as with 48-h *P. putida* biofilm the detached biofilm cells were also susceptible to the same concentration of GO showing similar reduction in viability (~ 3-log) after 24 and 48 h. In addition, GO was tested against planktonic *P. putida* and viability was assessed over time (Fig. 7). There was no significant change in viability of planktonic *P. putida* after GO treatment with a concentration ranging from 100 to 1600 μg/mL after 1, 2, 3, 4, and 5 days. These results further confirm that only 48-h *P. putida* biofilm cells were susceptible to GO-120.

Antibacterial studies of graphene materials have mainly focused on modifying their chemistry, but the physiological state of bacteria as a factor in susceptibility was not investigated. Biofilm formation is regulated by genetic and environmental factors and occurs through several developmental stages (Acemel et al. 2018). In each stage, the bacterial cells physiologically differ from cells in the other stages and biofilm cells may differ phenotypically from planktonic cells (Bester et al. 2005). The results in this study are in contrast to the belief that planktonic bacteria are more sensitive than biofilms. During the early stages of *P. putida* biofilm development (1 to 3 days), cells are non-motile and sessile inside the micro-colonies. After 3 days of growth, the micro-colonies reach a critical size and the bacteria start to swim rapidly in circles and the compact micro-colonies are dissolved to begin the formation of loose structures containing bacteria from different micro-colonies (Tolker-Nielsen et al. 2000). Actively dividing or exponentially growing bacteria are more susceptible to antibiotics compared with non-dividing or stationary phase bacteria (Mascio et al. 2007). After 48 h of maturation the cells were likely to be more actively dividing and at this stage may become susceptible to GO. However, further work is required (e.g. gene expression analysis) to understand the physiological state of *P. putida* cells in biofilm at different developmental stages.

Also, the production of EPS leads the micro-colonies to maturation, making the highly ordered structure of the biofilm. In *P. putida* biofilm, the EPS is comprised of polysaccharides (e.g. cellulose), xanthan, dextran (Camesano and Abu-Lail 2002), alginate (Chang et al. 2007), and proteins (e.g. LapA protein, a cell-to-surface adhesin) (Klausen et al. 2006). Also, *P. putida* has been reported to produce substantial amounts of extracellular DNA in the sessile mode of growth (Steinberger and Holden 2005). In this study the thickness of the EPS in biofilms without GO treatment increases gradually throughout the maturation stages of the biofilms from 24, 48, to 72 h (Fig. S9) but the viability of *P. putida* cells only significantly (*P* < 0.05) increased from 24 to 48 h while no significant increase occurred from 48 to 72 h (Fig. 2). From these results, it can be deduced that at 48 h, a lot of EPS constituents are being secreted from the cellular membrane in addition to still being actively dividing which may increase their susceptibility to GO. Moreover, although EPS constituents may still be secreted in 72-h biofilms, *P. putida* cells were non-dividing and have reached a stationary phase of growth which probably reduces their susceptibility to GO.

## Conclusion

Antibacterial activity of GO against biofilms was observed and this activity only occurred at a specific stage of biofilm maturity. GO was found to have antibacterial effect against 48-h biofilms, but no effect was observed against 24- and 72-h biofilms and cells detached from 24- and 72-h biofilms or planktonic cells. Moreover, similar trends were observed when the GO was tested against *P. putida* cells detached from 48-h biofilm. This age-related susceptibility to GO may be linked to the physiological state of the cells which differs at each maturation stage. For example, gene expression or secretion of molecules by the bacteria at a certain stage of biofilm maturity may be responsible for the observed susceptibility to GO, however, further investigation is required to fully understand their link. This may explain the inconsistencies of GO activity against bacteria reported in the literature. As the findings suggested that oxidative stress may be the mechanism behind the observed antibacterial activity of GO against 48-h biofilms, oxidative stress could be measured and compared between 24-, 48-, and 72-h biofilms in future work. The findings indicate important implications of GO accumulation for environmental systems where biofilm maturity varies.

## Electronic supplementary material

ESM 1 (DOCX 1975 kb)
