# Supplementary material for: Antibacterial effect of graphene oxide (GO) nano-particles against Pseudomonas putida biofilm of variable age
Source: Environ Sci Pollut Res Int. 2019 Jun 27;26(24):25057–70. doi: 10.1007/s11356-019-05688-9 (PMC6689283; doi:10.1007/s11356-019-05688-9)
Supplement: Supplementary file 1 — (DOCX 1975 kb) [file 11356_2019_5688_MOESM1_ESM.docx]

**Supplementary Material**


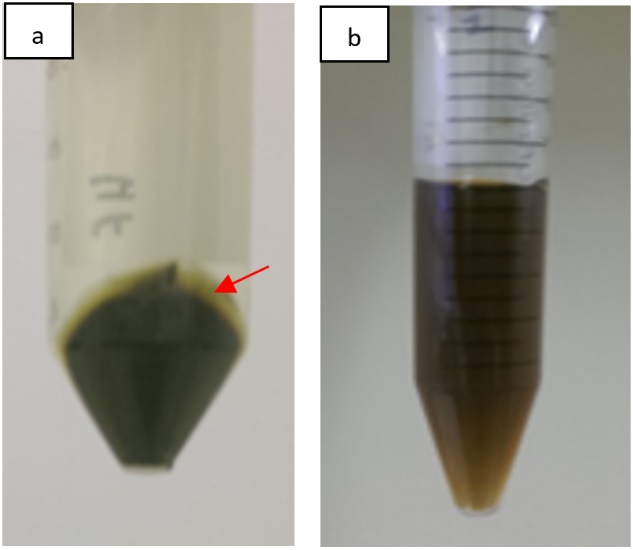


**Fig. S1** Graphene oxide purification steps (washings) before (A) and after separating from graphite (B). GO obtained using the modified Hummers’ method critically depends on the separation of a brownish/golden gel-like layer (GO) from a thick black sediment (graphite) (A) as a layer of GO appears when pH became neutralised after several washings.


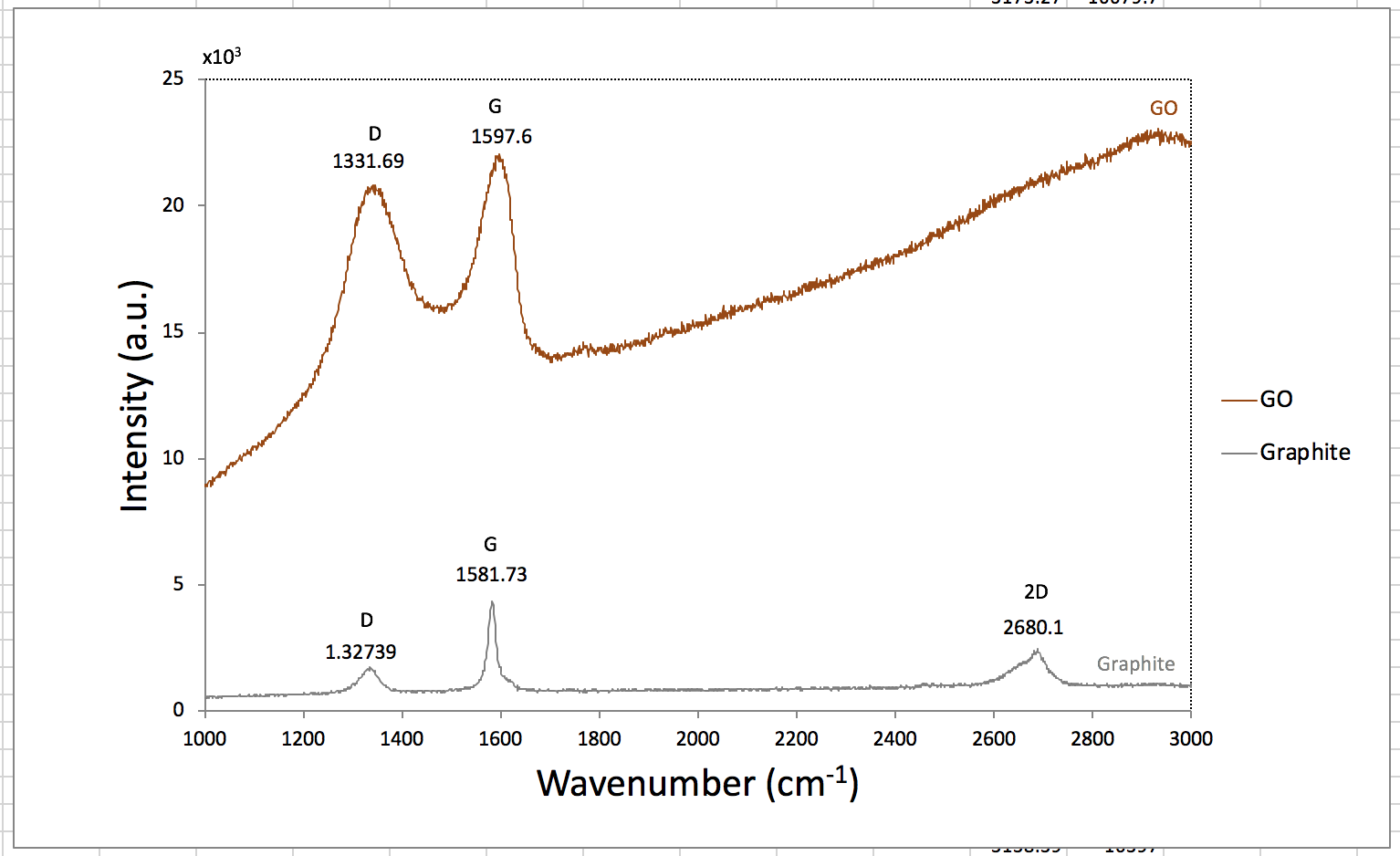


**Fig. S2** Raman spectra with D, G and 2D-band after the oxidation an exfoliation processes for graphite and GO. The spectra correspond to an exciting laser wavelength of 633 nm laser. GO and graphite both exhibited the D- and G-band. The D-band assigns to the breaking mode of *K-*point phonons with A_2g_ symmetry and the G-band attributes to the tangential stretching mode of the E_2g_ phonon of the carbon sp2 atoms. Graphite also shows a prominent 2D-band is a second-order two-phonon process which that was not found in GO.


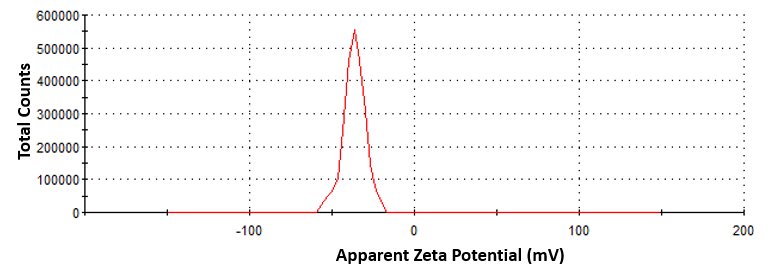


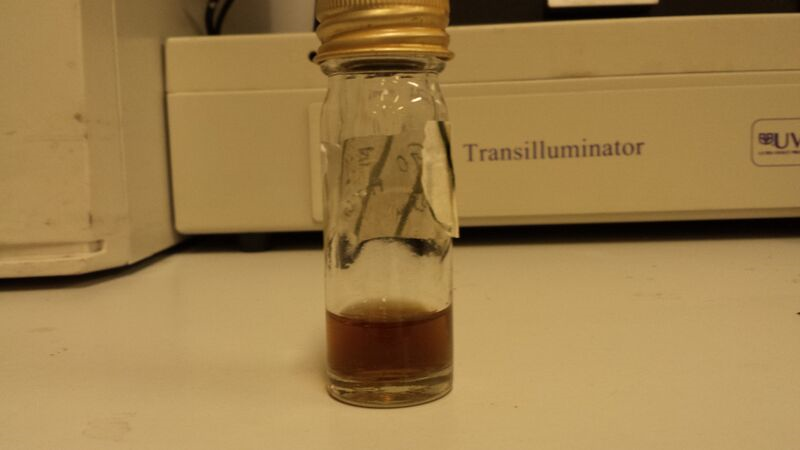

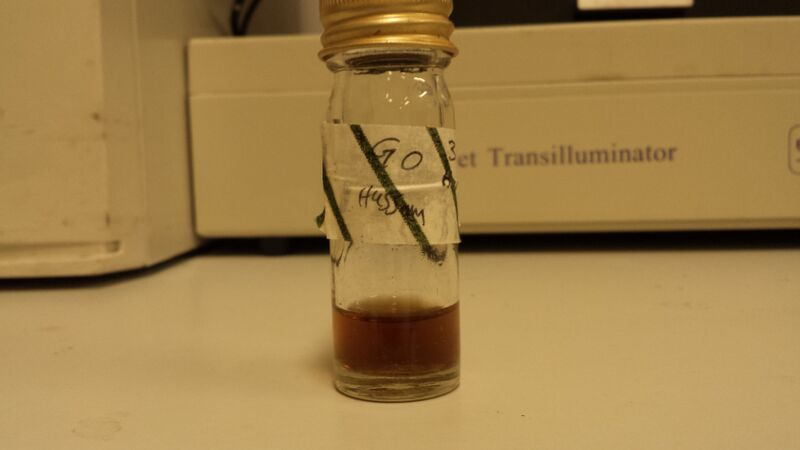


**Fresh GO-120**

**6 months** old GO-120

**b**

**a**

**Fig. S3** Stability of GO-120 sheets dispersed in water. Surface charge data of GO expressed as Zeta potential values from Zetasizer ζ = -34.6 ± 0.9 mV (A) and photographic images of fresh dispersion of GO-120 immediately after the reaction and after 6 months storage at ambient temperature (B).


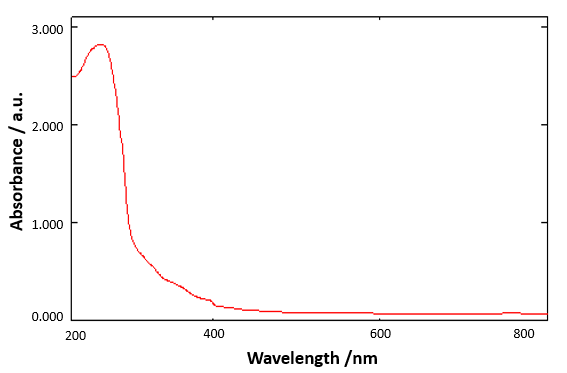


**Fig. S4** UV-Vis absorption spectra of GO exhibit a maximum absorption peak at about 235 nm, corresponding to a 𝜋-𝜋* of transition of aromatic C-C bonds.


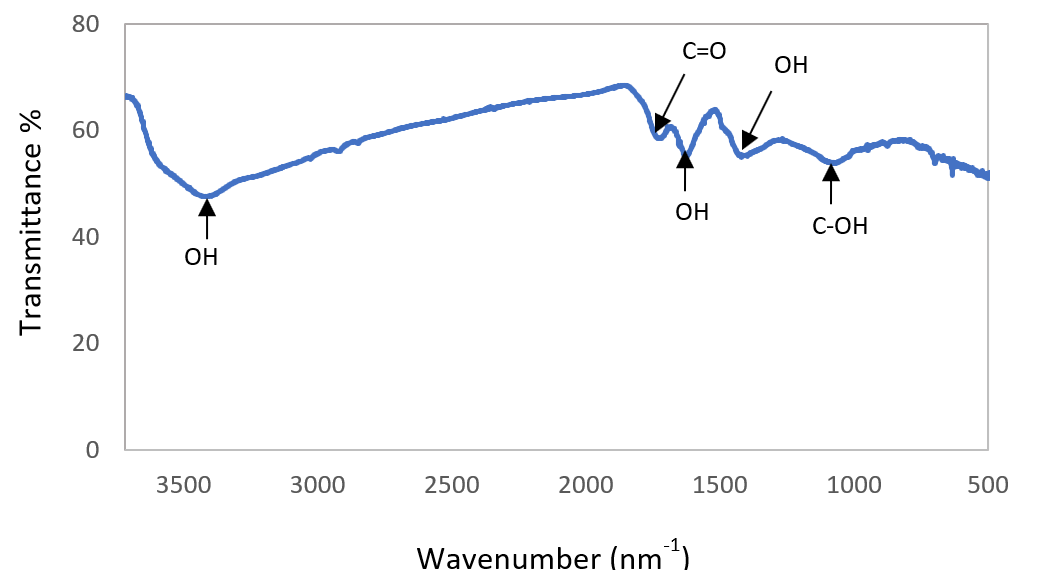


**Fig. S5** FT-IR spectra of GO-120 in the full range of wavenumbers.


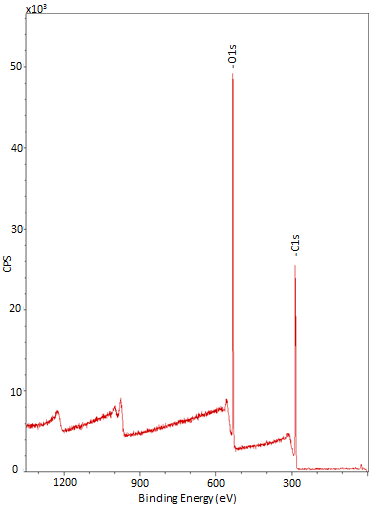

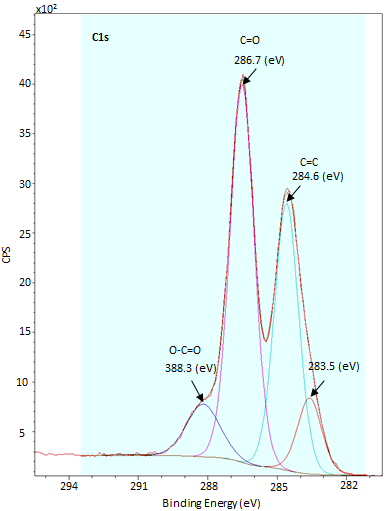


**b**

**a**


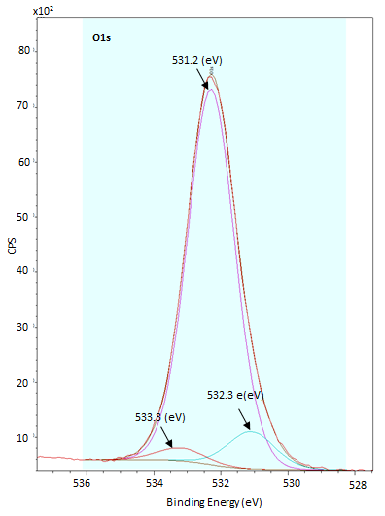


**c**

**Fig. S6** XPS result for GO-120 Chemical contamination by XPS survey spectra (A); and quantification of functional groups by high resolution C1s XPS spectra (B) and O1s XPS spectra (C).


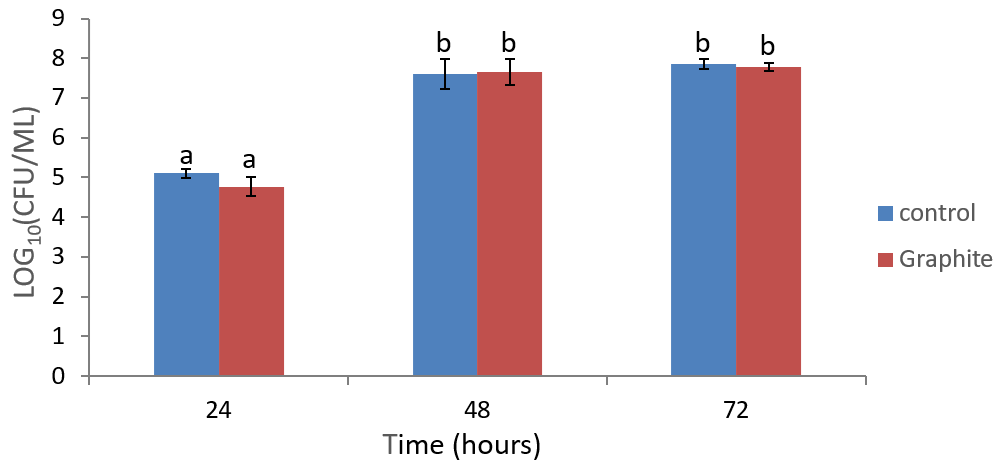


**Fig. S7** Viability of *P. putida* biofilm incubated with graphite flakes (85 µg/mL) for 24, 48 and 72 hours. Bars represent mean ± SEM taken from a minimum of 3 independent experiments. Mean values with different letters are significantly different (P < 0.05). The data was analysed with one-way ANOVA.


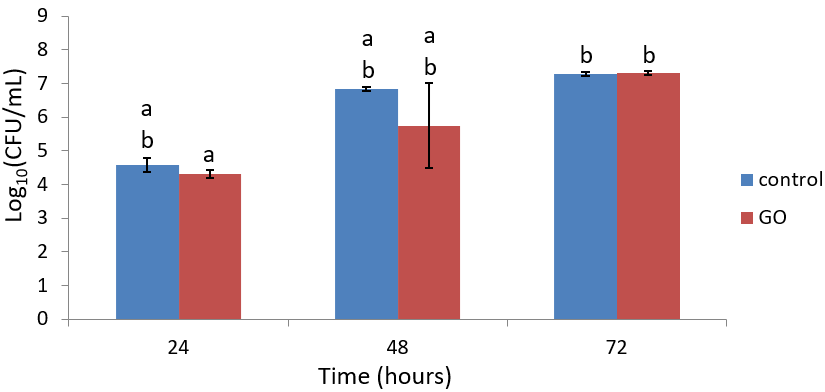


**Fig. S8** Viability of *P. putida* biofilm incubated with GO-120 (8.5 µg/mL) for 24, 48 and 72 hours. Bars represent mean ± SEM taken from a minimum of 3 independent experiments. Mean values with different letters are significantly different (P < 0.05). The data was analysed with one-way ANOVA.


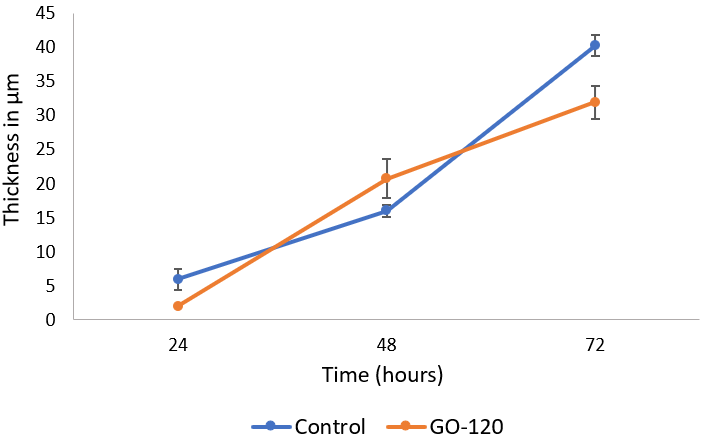


**Fig. S9** Thickness in µm of 24, 48 and 72-hour *P. putida* biofilm after GO-120 (85 µg/mL) treatment. Bars represent mean ± SEM taken from a minimum of 3 independent experiments.
